# MiR-145 inhibits the epithelial-to-mesenchymal transition via targeting ADAM19 in human glioblastoma

**DOI:** 10.18632/oncotarget.21442

**Published:** 2017-09-30

**Authors:** Xingqiang Wang, Enqin Wang, Jun Cao, Feng Xiong, Yonglin Yang, Haitao Liu

**Affiliations:** ^1^ Department of Neurosurgery, Rizhao People's Hospital, Jining Medical University, Rizhao 276826, Shandong, China; ^2^ Clinical Skill Training Center, Rizhao People's Hospital, Jining Medical University, Rizhao 276826, Shandong, China

**Keywords:** MiR-145, ADAM19, GBM, EMT

## Abstract

In recent years, increasing studies demonstrated that miR-145 plays a tumor suppressor role in many human cancers. In the present study, we evaluated the expression of miR-145 and A Disintegrin and Metalloproteinase 19 (ADAM19) in glioblastoma multiforme (GBM) tissues and cells. Furthermore, we investigated the mechanisms underlying miR-145/ADAM19-induced GBM biology. Here, we found that miR-145 expression was down-regulated, while ADAM19 expression was up-regulated in GBM tissues and cells. Moreover, miR-145 mimics repressed U87 and U251 cell proliferation, migration and invasion. miR-145 mimics also inhibited the epithelial-to-mesenchymal transition (EMT) of U87 and U251 cells. Mechanically, the 3′ untranslated region (3′-UTR) of ADAM19 mRNA was a direct target for miR-145. In addition, ADAM19 over-expression also partially abrogated miR-145-inhibited EMT. In conclusion, this work suggested that high miR-145 expression inhibited EMT of GBM cells by targeting ADAM19. Thus miR-145/ADAM19 can be suggested as a novel target for GBM patients.

## INTRODUCTION

Human brain glioma accounts for a half of malignant brain tumors, which is aggressive, invasive and hard to treat [1, 2]. Patients with gliomas usually suffer from surgical operation combined with radio-chemotherapy, particularly those with GBM, who had a very short survival time (less than 2 years) [3, 4]. Overall, the death rate of GBM patients within 3 years of diagnosis exceeds 90%, and patients with recurrent GBM had a poor prognosis [5, 6]. According to recent reports, the development and progression of glioma is closely related to imbalance of oncogenes and tumor-suppressing genes. Thus, it is essential to find a novel therapeutic approach for GBM patients.

MiRNAs are a kind of non-coding small RNAs, and have 19∼23 nucleotide sequences. As reported, miRNAs play an essential role in cell differentiation, proliferation, and apoptosis by inhibiting or promoting the post-transcription mRNA expression [7, 8]. In addition, miRNAs also induces the degradation of mRNAs at a post-transcriptional level, and affects initiation of translation via binding to the 3′-untranslated regions of target mRNAs [9]. These studies suggested that miR-145 might be an effective and useful way to treat human tumors [10, 11]. Besides, miR-145 also serves as a new biomarker for colorectal cancer [12], pancreatic cancer [13] and breast cancer [14, 15]. However, the specific role of miR-145 in the development of GBM is still unclear.

In our work, we investigated the impact of miR-145 on human GBM. Firstly, we detected the expression level of miR-145 and ADAM19 in human GBM cells and tissues. Secondly, we explored cell cycle, invasion, and migration. Finally, we explored whether the 3′-UTR of ADAM19 mRNA was a direct target for miR-145.

## RESULTS

### MiR-145 expression level in GBM tissues and cells

**Figure 1:**
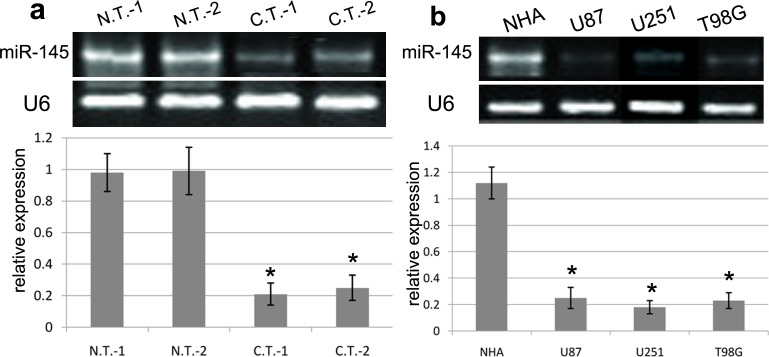
The expression of miR-145 in GBM tissues and cell lines **(a)** The RT-PCR analysis of miR-145 expression was conducted in representative 2 cases of GBM tissues (C.T.) and matched normal tissues (N.T.). **(b)** The RT-PCR analysis of miR-145 expression was conducted in NHA, U87, U251 and T98G cell lines. Quantification analysis was defined as the relative density of miR-145 to U6. U6 was used as an internal control. Results shown are the mean ± SEM of repeated independent experiments. ^*^p<0.001, compared with control, one-way ANOVA.

To explore the biological effect of miR-145 in the development of GBM, we carried out RT-PCR assay using GBM tissues and cells. We found that the expression of miR-145 was significantly down-regulated in two cases of GBM tissues as compared with normal brain tissues (Figure 1a). In addition, the expression of miR-145 was obviously decreased in U87, U251 and T98G cell lines in comparison with normal NHA cells (all p<0.001) (Figure 1b). In general, the results from 30 cases of glioblastoma tissues revealed that miR-145 expression was significantly reduced in the GBM tissues in comparison with normal tissues (p<0.001). Thus, our findings suggested that miR-145 is closely related to the development of human GBM.

### ADAM19 expression in GBM tissues and cells

**Figure 2:**
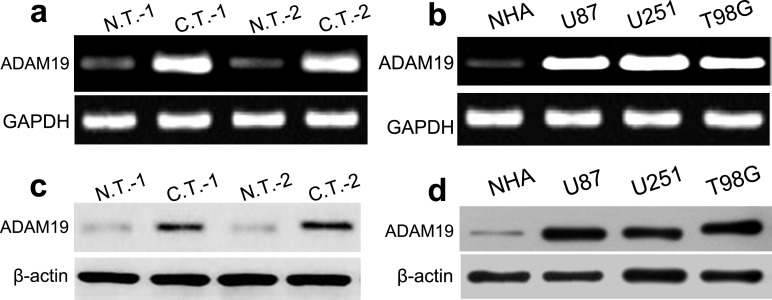
ADAM19 is increased in GBM tissues and cell lines **(a-b)** The mRNA expression of ADAM19 was higher in GBM tissues and cells lines (U87 and U251, and T98G) compared with in NHA cells using RT-PCR. **(c-d)** The protein expression of ADAM19 was higher in GBM tissues and cells lines (U87, U251 and T98G) compared with that in NHA cells using western blot.

Next we explored the expression of ADAM19 in GBM tissues and cells using RT-PCR and western blot assays. RT-PCR analysis revealed that ADAM19 mRNA was significantly highly expressed in two cases of GBM tissues as compared with normal brain tissues (Figure 2a). ADAM19 mRNA expression was also obviously increased in U87, U251 and T98G cell lines as compared with normal NHA cells (all p<0.001) (Figure 2b). In addition, western blot assay revealed that the expression of ADAM19 protein was significantly up-regulated in two cases of human GBM tissues, U87, U251 and T98G cell lines respectively (all p<0.001) (Figure 2c, 2d). According to statistics, we demonstrated that the expression level of miR-145 was inversely proportional to that of ADAM19 mRNA (r=-0.821, p=0.005). Likewise, the expression level of miR-145 was inversely proportional to that of ADAM19 protein (r=-0.810, p=0.003). These results indicated that the expression of ADAM19 is also involved in the development of human GBM.

### miR-145 inhibits U87 and U251 cell cycle

**Figure 3:**
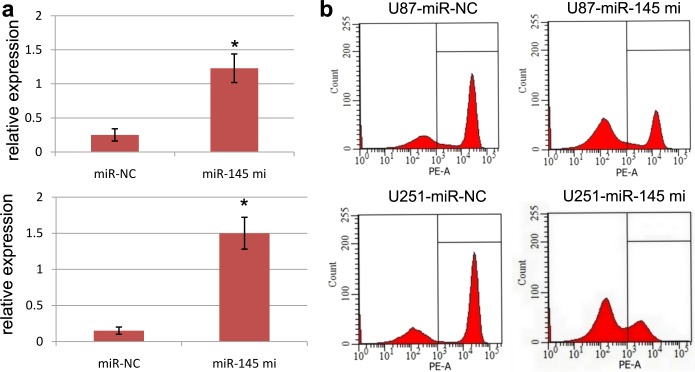
Ectopic miR-145 expression affects cell cycle **(a)** The U87 and U251 cells transfected with miR-145 mimics showed high expression of miR-145 using RT-PCR assays. ^*^p<0.001, compared with control, one-way ANOVA. **(b)** Flow cytometry assays showed a statistically significant increase or decrease in cell proliferation when miR-145 was overexpressed in U87 and U251 cells. Results shown are the mean ± SEM of repeated independent experiments. ^*^p<0.001, compared with control, one-way ANOVA.

To investigate the potential role of miR-145 in U87 and U251 cell proliferation, we transfected miR-145 mimics or miR-NC into U87 and U251 cells. miR-NC was used as control. Firstly, we demonstrated that the expression of miR-145 was obviously increased in U87 and U251 cells according to RT-PCR analysis (Figure 3a). Secondly, we carried out flow cytometry to investigate the potential role of miR-145 in GBM cell cycle. Flow cytometry analysis revealed that the proportion of U87 and U251 cells in G0/G1 phase in the control group was 42.5% and 41.9% respectively. However, the proportion of U87 and U251 cells in G0/G1 phase was obviously increased (75.4% and 83.5%) than control with significant differences (both p<0.05) (Figure 3b). These results indicated that miR-145 inhibits U87 and U251 cell proliferation via regulation cell cycle.

### miR-145 represses GBM cell migration and invasion

**Figure 4:**
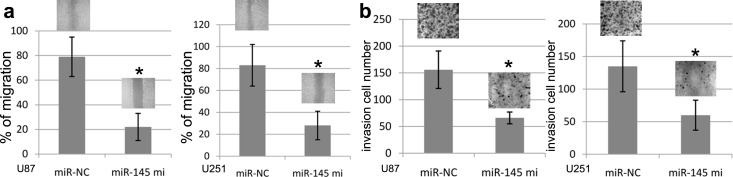
miR-145 expression and U87 and U251 cell migration and invasion Increasing miR-145 expression in U87 and U251celllines suppressed cell migration and inhibited cell invasion. **(a)** In the wound healing assay, U87 and U251 cell lines transfected with miR-145 or miR-NC were seeded in the 6-well dishes, and a scratched wound was applied at 24 hours post-transfection. Cell migration to the scratched wound was measured at 0 and 24 hours and compared between cells transfected with miR-145 and miR-NC. **(b)** In the transwell invasion assay, U87 and U251 cell lines transfected with miR-145 or miR-NC were seeded to the Matrigel coated invasion upper chambers at 24 hours post-transfection and allowed to invade toward the other side of chambers for 20 hours. The invading cellsunderneath the chambers were stained and counted. Representative images under microscopy from different experimental conditions were taken from each cell line. All histograms show invading cell numbers per microscopic field from three independent experiments. ^*^p<0.001, compared with NC miRNAs, one-way ANOVA.

To elucidate the impact of miR-145 on U87 and U251 cell migration and invasion, we transfected miR-145 mimics or miR-NC into U87 and U251 cells, and then all cells were subjected to the wound healing and transwell assays. In this study, the wound healing assay validated that ectopic miR-145 expression induced by miR-145 mimics obviously inhibited the wound width of U87 and U251 cell than control (Figure 4a). At the same time, the transwell assay validated that ectopic miR-145 expression induced by miR-145 mimics obviously inhibited the invasion number of U87 and U251 cells as compared with miR-NC (Figure 4b).

### miR-145 represses EMT of GBM cells

**Figure 5:**
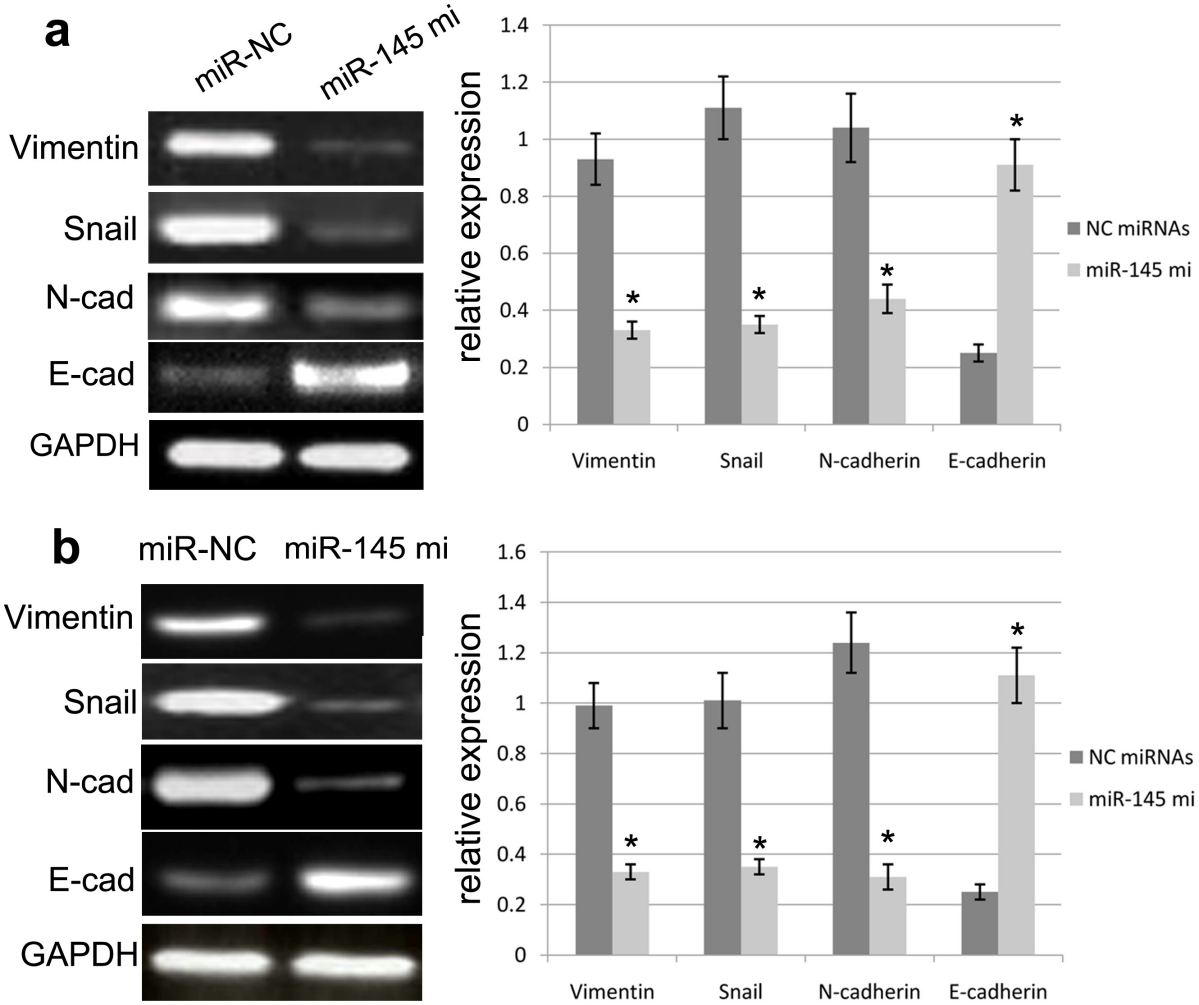
miR-145 affected the mRNA expression of EMT biomarkers miR-145 overexpression can enhance the E-cadherin expression and repress the expression of N-cadherin, Vimentin and Snail in U87 **(a)** and U251 **(b)** cells as determined by Western blot. Quantification was normalized to GAPDH. GAPDH was used as an internal control. Results shown are the mean±SEM of three repeated independent experiments. ^*^p<0.001, compared with control, one-way ANOVA.

**Figure 6:**
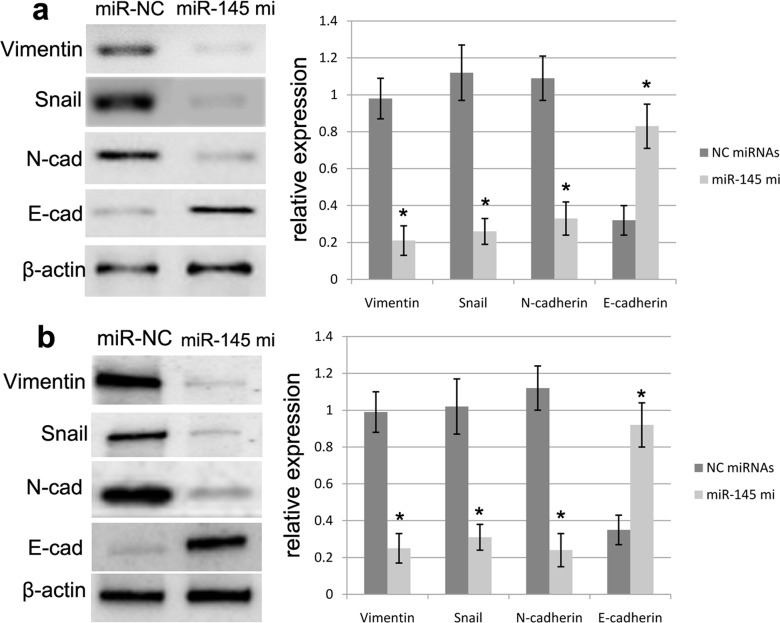
miR-145 affected the protein expression of EMT biomarkers miR-145 overexpression can enhance the E-cadherin expression and repress the expression of N-cadherin, Vimentin and Snail in U87 **(a)** and U251 **(b)** cells as determined by Western blot. Quantification was normalized to β-actin. β-actin was used as an internal control. Results shown are the mean±SEM of three repeated independent experiments. ^*^p<0.001, compared with control, one-way ANOVA.

To further figure out the effect of miR-145 over-expression on EMT of U87 and U251 cells, we detected the mRNA and protein expression changes of EMT biomarkers, including N-cadherin, vimentin, Snail and E-cadherin in U87 and U251 cells transfected with miR-145 mimics or miR-NC. RT-PCR assay revealed that ectopic miR-145 expression mediated by miR-145 mimics promoted the expression of E-cadherin protein, and repressed the expression of N-cadherin, vimentin and Snail in U87 and U251 cells (Figure 5). Moreover, western blot assay revealed that ectopic miR-145 expression mediated by miR-145 mimics the E-cadherin protein expression, and suppressed the expression of N-cadherin, vimentin and Snail (Figure 6). Our results validated that over-expression of miR-145 indeed affects EMT progression of U87 and U251 cells.

### ADAM19 3′-UTR is a direct target of miR-145

**Figure 7:**
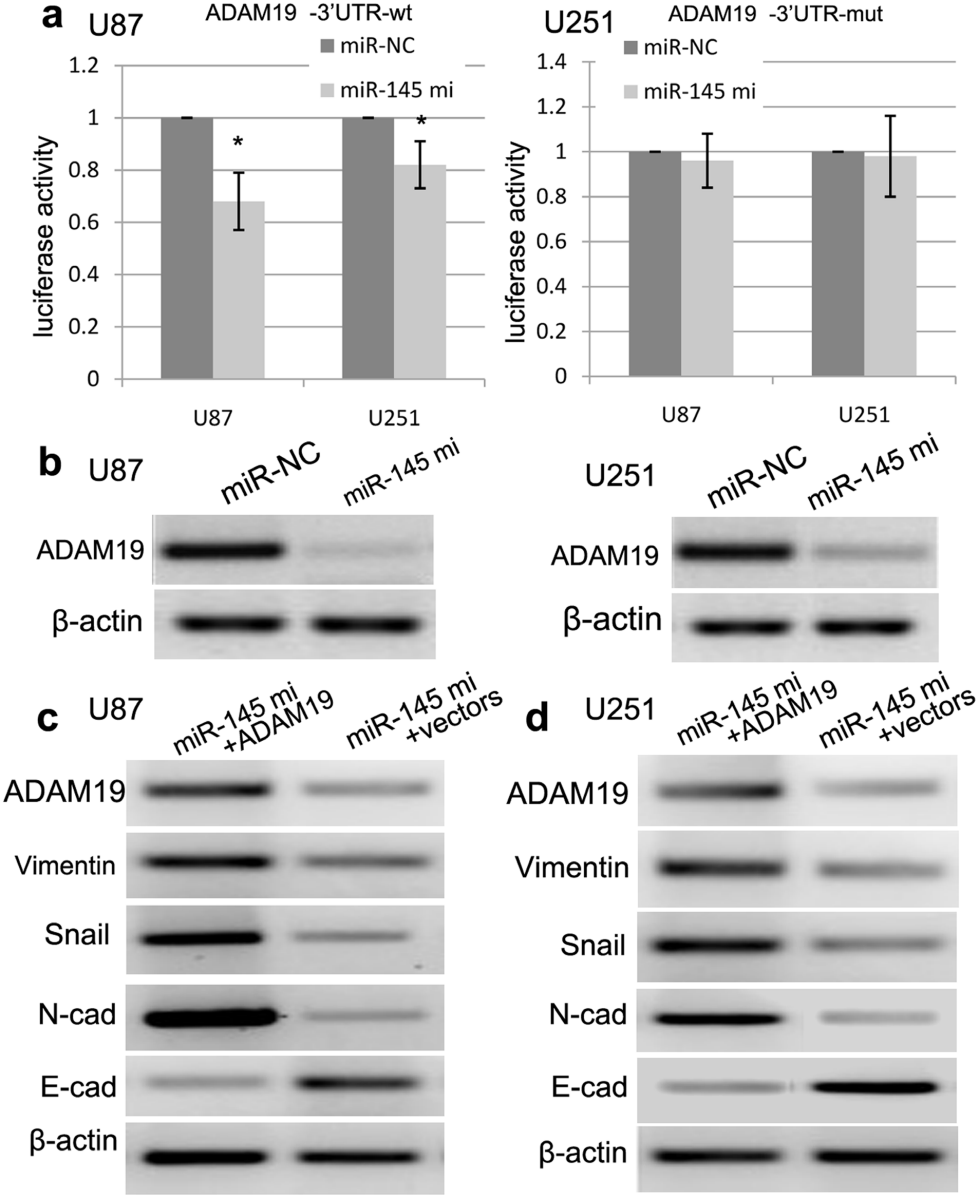
miR-145 regulates ADAM19 expression **(a)** The sequences of miR-145 binding sites within the human ADAM19 3′-UTRs and schematic reporter constructs, in this panel, ADAM19-wt represent the reporter constructs containing the entire 3′-UTR sequences of ADAM19. ADAM19-mut represent the reporter constructs containing mutated nucleotides. miR-145 significantly suppressed the luciferase activity that carried 3′-UTRwt but not 3′-UTR-mut of ADAM19. **(b)** Overexpression of miR-145 reduced the expression of ADAM19 in U87 and U251 cells. The expression of ADAM19 was normalized to β-actin. **(c)** Ectopic expression of ADAM19 can decrease the E-cadherin expression, and increase the expression of N-cadherin, Vimentin and Snail using RT-PCR. **(d)** Ectopic expression of ADAM19 can decrease the E-cadherin expression, and increase the expression of N-cadherin, Vimentin and Snail using Western blot. Quantification was normalized to β-actin. β-actin was used as an internal control. Results shown are the mean±SEM of three repeated independent experiments. ^*^p<0.001, compared with control, one-way ANOVA.

To confirm whether miR-145 directly binds to the 3′-UTR of ADAM19 mRNA, we cloned a full-length 3′-UTR (wt/mut) of ADAM19 mRNA, which was inserted into a luciferase reporter vector with downstream from the firefly luciferase gene. In this study, western blot was used to detect the expression level of ADAM19 protein. The luciferase reporter assay revealed that miR-145 mimics-induced miR-145 overexpression obviously decreased the luciferase activity of ADAM19-3′UTR-wt as compared with control (p<0.001) (Figure 7a). By contrast, miR-145 mimics did not affect the luciferase activity of ADAM19-3′UTR-mut (Figure 7a). Besides, western blot assay further revealed that the expression of ADAM19 protein was obviously decreased in U87 and U251 cell lines co-transfected with miR-145 mimics and ADAM19-3′-UTR-wt as compared with those with miR-145 mimics and ADAM19-3′UTR-mut (Figure 7b).

### ADAM19 restoration reverses miR-145-inhibited EMT

To further demonstrate the involvement of ADAM19 in miR-145-induced EMT, we tried to up-regulate the expression of ADAM19 in U87 and U251 cells with miR-145 mimics using transfection of pCDNA-ADAM19 plasmids. As expected, we found that the expression of ADAM19 was increased in U87 and U251 cells transfected with pCDNA-ADAM19 plasmids, and ADAM19 plasmids can decrease the expression of E-cadherin, and increase the expression of N-cadherin, Vimentin and Snail (Figure 7c, 7d), indicating that miR-145 has a negatively regulatory effect on ADAM19 in U87 and U251 cells.

## DISCUSSION

In recent years, miR-145 has been reported to play an important role in regulating normal cellular functions, while the deregulated miRNAs expression might result in the initiation of tumors and malignant progression [12–14]. In addition, ADAM19, a member of a disintegrin and metalloproteinases, are implicated into many biological and pathological processes, including embryonic development, cell growth, migration, and invasion [16, 17]. In the present study, we evaluated the expression of miR-145 and ADAM19 in GBM tissues and cells. Furthermore, we investigated the mechanisms underlying miR-145/ADAM19-induced GBM biology.

In this work, we found that the expression of miR-145 was significantly down-regulated in GBM tissues as compared with normal brain tissues. In addition, the expression of miR-145 was obviously decreased in U87, U251 and T98G cell lines in comparison with normal NHA cells. Meanwhile, RT-PCR analysis revealed that ADAM19 mRNA was significantly highly expressed in GBM tissues as compared with normal brain tissues. ADAM19 mRNA expression was also obviously increased in U87, U251 and T98G cell lines as compared with normal NHA cells. Consistent with reports, Mo D et al reported that miR-145 expression was diminished in lung adenocarcinom, and obviously related to the lymph node metastasis, and was negatively associated with N-cadherin mRNA expression [18]. Li Y et al demonstrated that miR-145 expression was downregulated in osteosarcoma tissues, and was remarkably correlated with the occurrence of aggressive osteosarcoma [19]. These studies indicated that miR-145 was involved into the development of GBM.

In the present study, miR-145 over-expression repressed U87 and U251 cell proliferation, migration and invasion, indicating that miR-145 might affect EMT. It has been reported that EMT plays an essential role in GBM cells migration and invasion [20, 21]. When EMT occurs, cancer cells exhibited the decrease of cellular adhesion protein E-cadherin, and the increase of mesenchymal biomarkers N-cadherin, Snail and vimentin. In addition, a variety of molecules in traditional signaling pathways were involved into EMT. Our study demonstrated that up-regulation of miR-145 facilitated the expression E-cadherin, and reduced the expression of N-cadherin, Vimentin and Snail, indicating that miR-145 indeed regulated EMT.

Mechanically, miRNAs also induces the degradation of mRNAs at a post-transcriptional level, and affects initiation of translation via binding to the 3′-untranslated regions of target mRNAs [9]. We also found that miR-145 mimics obviously decreased the luciferase activity of ADAM19-3′UTR-wt. By contrast, miR-145 mimics did not affect the luciferase activity of ADAM19-3′UTR-mut. Notably, over-expression of ADAM19 did not totally abrogate miR-145-inhibited EMT, indicating that there exist some other potential targets of miR-145. Consistent with reports, Sathyanarayanan A et al found that ectopic miR-145 expression or SIP1 siRNAs inhibited C33A and SiHa cell proliferation, migration and invasion, and altered the expression of EMT-associated biomarkers, and miR-145 could inhibit the expression of the SMAD-interacting protein 1 in human cervical cancer cells [22]. Han Q also demonstrated that miR-145 can specifically bind to the 3′-UTR of the connective tissue growth factor and markedly inhibited the luciferase activity, and repressed the proliferation, migration, invasion, and EMT of esophageal squamous cell carcinoma cells [23]. These findings revealed that miR-145 expression exerts a vital effect on GBM progression.

In conclusion, miR-145 inhibited GBM cell proliferation, migration, invasion and EMT. This work suggested that high miR-145 expression inhibited EMT of GBM cells by targeting ADAM19. Thus miR-145/ADAM19 can be suggested as a novel target for GBM patients.

## MATERIALS AND METHODS

### Ethics statement

The present study was approved by the Ethics Committee of Ethics Committee of Rizhao People's Hospital. Patients enrolled in this study signed written informed consent. All procedures were subjected to the Declaration of Helsinki.

### Patients and samples

Human tissue acquisition and use in this study complied with the National Regulations on the Use of Clinical Samples in China. 30 cases of tissue specimens were obtained from archived tissue samples from patients with GBM who underwent surgical treatment at Rizhao People's Hospital from January 2014 to December 2016. Before the RNA extraction from frozen tissues, the adjacent tumor tissues were subjected to frozen sections and reviewed by a pathologist to ensure that a minimum of 80% tumor cells were included in the sample. For GBM patients, none of them had received chemotherapy or radiotherapy prior to surgery, and all patients were well followed up. Patients, who died of diseases not directly related to their gliomas or due to unexpected events, were excluded from this study.

### Cell culture

As cell culture, primary normal human astrocytes (NHA) were purchased from the Sciencell Research Laboratories (Carlsbad, CA) and cultured under the conditions as instructed by the manufacturer. The human GBM cell lines used in this study included U87 and U251 and T98G. These cell lines were obtained from the ATCC (Rockville, USA). Cells were kept in Dulbecco's modified Eagle's medium (Gibco; Invitrogen) supplemented with 10% fetal bovine serum (GIBCO, NY, USA), streptomycin and penicillin at 37°C in a humidified 5% CO_2_ incubator. Cells were passaged every 5 days by washing twice in 5 mL phosphate-buffered saline (PBS), then incubating with 0.25% trypsin. In addition, normal glial cell NHA was also included as control.

### Cell transfection

In this study, pCDNA-ADAM19 plasmids, pCDNA-vector plasmids, miR-145 mimics (50 nM) and NC miRNAs were purchased from Ambion (Austin, TX). Oglionucleotides with scramble sequence without any known target in human genome was used a control of miRNAs or siRNA. Scramble sequence with fluorescence was used to determine the transfection efficiency. The empty pmirGLO vector without any insert was used as a control of plasmid transfection. Lipofectamin 2000 (Invitrogen, Carlsbad, CA) was used for the transfection.

### Western blot analysis

Cells were plated at a density of 1×10^5^ cells/well in six-well plates. At 24 hours after treatment, the cells were washed with ice-cold PBS and lysed with lysis buffer. The cell lysate (50 μg protein) was subjected to 10% sodium dodecyl sulfate-polyacrylamide gel electrophoresis, and the protein bands were transferred to a polyvinylidene difluoride membrane. The membrane was blocked with Tris-buffered saline containing 0.1% Tween 20 and 5% nonfat, dry milk for 1 hour, and then incubated overnight at 4°C with the primary antibody (antibody, ADAM19, Vimentin, Snail, N-cadherin and E-cadherin) (Santa Cruz) (1: 1,000). The membrane was washed three times with PBS containing 0.1% Tween 20, and incubated with horseradish peroxidase-conjugated anti-rabbit immunoglobulin G as the secondary antibody (1:1000) for 1 hour at room temperature. Protein bands were detected using the enhanced chemiluminescence system and analyzed using densitometry. Quantitative analysis was performed with β-actin as an internal control.

### RNA extraction and RT-PCR

Trizol reagent (Invitrogen, USA) was used to get total RNA from tissues or cells. The sequences of primersused in this study were

miR-145 primers: forward 5′-GCTAGCTCGTACC GTTAATAA-3′, reverse 5′-ATTCTAGAGGCCGAGGCGGCCGACATGT-3′;

ADAM19 primers: forward 5′-ATGGCGAGTGT AGTGCTGC-3′; reverse 5′-GATCACTGTCACGTCT TTCGT-3′;

U6 primers: forward 5′-CTCGCTTCGGCAGCA CA-3′, reverse 5′-AACG CTTCACGAATTTGCGT-3′

GAPDH primers: forward 5′-GCACC GTCAAGGCTGAGAAC-3′, reverse 5′-TGGTGAA GACGCCAGTGGA-3′

Total RNA (1 μg) was reverse-transcribed into cDNA using a Quantscript RT Kit (Tiangen, China). PCR was performed using a PCR MasterMix Kit (BioTeke, China) in a GeneAmp PCR system 9600 (ABI Int.). cDNA was amplified under the thermocycling conditions as follows: 3 min initial denaturation at 94°C (1 cycle), 30 s denaturation at 94°C (35 cycles), 30 s annealing at 57°C, and 45 s extension at 72°C. The last amplification was followed by final 7 min incubation at 72°C. PCR products were separated by electrophoresis through 1% agarose gel, stained with ethidium bromide, and visualized by UV transillumination in a Tocan Gel Imaging System (Tocan Co., Shanghai, China). GAPDH was used as an internal control. The mRNA level was calculated by determining the integrated intensity of the bands of each treated group as a ratio of the control. Each sample was measured in triplicate. The gene expression threshold cycle values of miRNAs were calculated by normalizing with internal control RNU6 and relative quantization values were calculated.

### Wound healing and invasion assay

An artificial wound was created by scraping with a micropipette tip. Then it was washed to remove dislodged cells, and was cultured in growth medium. To visualize migrated cells and wound healing, images were taken at 0 hour and 48 hours. For invasion assay, cells were added to the upper well chamber and coated with Matrigel (BD Biosciences) for 24 hours. Then, cells on the upper membrane surface were removed by careful wiping with a cotton swab, and the filters were fixed with 95% ethanol and stained with 0.2% crystal violet solution (Sigma) and then counted.

### Cell cycle analysis by flow cytometry

For cell cycle analysis, after treatment for 48 h, the cells were collected and then fixed with 70% ice-cold ethanol at 4°C for 24 h. The stationary liquid was removed through centrifugation, and then 100 μL of RNase A and 400 μL of PI were added, followed by incubation at room temperature for 30 min without light. Next, a FACSCalibur flow cytometer (Becton-Dickinson) equipped with a 488-nm argon laser was used, and cell cycle analysis was performed using Cell Quest software and ModFit.

### Dual luciferase assays

Cells were co-transfected with firefly luciferase reporter vector containing the ADAM19 3′-UTR-wt or 3′-UTR-mut. The control vector contained Renilla luciferase, pRL-TK (Promega) using lipofectamine 2000 (Invitrogen). Firefly and Renilla luciferase data were done with dual luciferase assays (Promega) after 48 hours of transfection.

### Statistical analysis

All data were analysed by SPASS 16.0, the data normality was verified by K-S test, and if the P value was more than 0.05, the data obeyed a normal distribution. The data were presented as the means ± SD for at least three independent experiments. One-way ANOVA and Student's t-test were performed for statistical analysis. A P value less than 0.05 was considered statistically significant.
